# Role of Cdc23/Mcm10 in generating the ribonucleotide imprint at the *mat1* locus in fission yeast

**DOI:** 10.1093/nar/gkz092

**Published:** 2019-02-13

**Authors:** Balveer Singh, Kamlesh K Bisht, Udita Upadhyay, Avinash Chandra Kushwaha, Jagpreet Singh Nanda, Suchita Srivastava, Jai Kumar Saini, Amar J S Klar, Jagmohan Singh

**Affiliations:** 1 Institute of Genetics and Development of Rennes, Faculte de Medecine, Campus santé de Villejean, 2 avenue du Professor Leon Bernard, CS 34317, 35043 Rennes Cedex, France; 2 Translational Discovery Biology, (Immuno-Oncology), Bristol-Myers Squibb Route 206 & Province Line Road, Princeton, NJ 08543, USA; 3 Department of Anesthesiology, RMSB 8022, 1600 NW, 10th Ave., Miami, FL 33136, USA; 4 Institute of Nano Science and Technology, Phase 10, Mohali 160062, USA; 5 Department of Pharmacology, Case Western Reserve University, 10900 Euclid Ave, Cleveland, OH 44106, USA; 6 QC Division, Central Research Institute, Kasauli, Himachal Pradesh 173204, India; 7 Institute of Microbial Technology, Sector 39A, Chandigarh 160036, India; 8 Gene Regulation and Chromosome Biology Laboratory, National Cancer Institute, Center for Cancer Research, National Institutes of Health, Building 539, Room 154, Frederick, MD 21702-1201, USA

## Abstract

The developmental asymmetry of fission yeast daughter cells derives from inheriting ‘older Watson’ versus ‘older Crick’ DNA strand from the parental cell, strands that are complementary but not identical with each other. A novel DNA strand-specific ‘imprint’, installed during DNA replication at the mating-type locus (*mat1*), imparts competence for cell type inter-conversion to one of the two chromosome replicas. The catalytic subunit of DNA Polymerase α (Polα) has been implicated in the imprinting process. Based on its known biochemical function, Polα might install the *mat1* imprint during lagging strand synthesis. The nature of the imprint is not clear: it is either a nick or a ribonucleotide insertion. Our investigations do not support a direct role of Polα in nicking through putative endonuclease domains but confirm its indirect role in installing an alkali-labile moiety as the imprint. While ruling out the role of the primase subunit of Polα holoenzyme, we find that mutations in the Polα-recruitment and putative primase homology domain in Mcm10/Cdc23 abrogate the ribonucleotide imprint formation. These results, while confirming the ribonucleotide nature of the imprint suggest the possibility of a direct role of Mcm10/Cdc23 in installing it in cooperation with Polα and Swi1.

## INTRODUCTION

**Figure 1.**
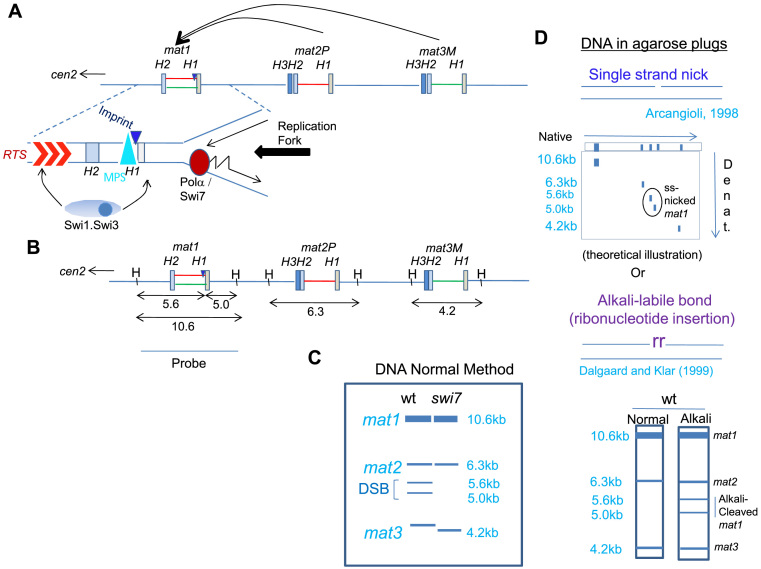
Schematic diagram depicting the organization of mating-type loci in *S. pombe*. (**A**) The loci *mat1, mat2* and *mat3* are located in ∼30kb region of chromosome 2. They comprise short conserved homology regions H1 to H3, which flank the ∼1.1 kb allele-specific sequences. Following imprinting at the boundary of *mat1* and the allele-specific region (dark blue triangle), a copy of the donor locus (*mat2* or *mat3*) is transposed to the *mat1* locus resulting in its switching by gene replacement. Replication fork progression from the centromere distal direction is met by a pause site (MPS1, blue triangle; 9), while fork from the left side encounters the replication termination sequence (RTS; 51). (**B**) A schematic diagram representing the *Hin*dIII restriction pattern of the mating type region with the corresponding result obtained following Southern blot hybridization using the *mat1P* or *M* fragment of 10.6 kb as a probe, schematically represented in (**C**), wherein *mat1, mat2* and *mat3* loci migrate at the positions of 10.6, 6.2 and 4.3 kb, respectively. Occurrence of imprint at *mat1* generates a fragile site, which appears as a double strand break (DSB) generating the bands of 5.6 and 5.0 kb when DNA is prepared by the conventional method (left lane). Due to lack of imprint no DSB is seen in *swi1, swi3* or *swi7* mutants. (**D**) Schematic representation of the methodology used to detect a nick as the imprint, which can be visualized by 2-dimensional gel electrophoresis. (Top panel) DNA is prepared in plugs and then resolved by acrylamide gel electrophoresis in the first dimension, while the 2^nd^ dimension is run in a denaturing acrylamide gel, as described earlier (13). Alternatively, samples embedded in agarose are digested with *Hin*dIII and then subjected to electrophoresis in native agarose gel without or with alkali-treatment (lower panel; 7), followed by Southern blotting and hybridization as in (**C**).

In *Schizosaccharomyces pombe*, the mating-type region comprises three loci located on chromosome II: *mat1M* or *P, mat2P* and *mat3M* (Figure 1A). The *mat1* cassette is expressed and it dictates the Plus or Minus sex/cell type to the cell. The *mat2P* and *mat3M* cassettes are transcriptionally silenced by an epigenetic mechanism and function as master copies for switching *mat1*. Switching occurs by highly regulated recombination through transposition/substitution of the *mat1* allele with the opposite mating-type information, copied from either *mat2P* or *mat3M* cassettes (1–4). The mating-type switching is an exquisitely regulated process. First, of the four ‘granddaughter’ cells derived from a single cell, only one cell switches in nearly 90% of pedigrees (2,5,6). This pattern results from asymmetric cell division occurring in each of the two consecutive generations in the progeny of a single cell. The switching program is initiated by a novel ‘imprint,’ that is installed in specific DNA strand during replication of the *mat1* locus on chromosome II (1,2). In the following cell division, the imprint consummates into a switch but only in one of the sister chromatids during *mat1* replication. The *mat1*-switching event removes the imprint. The *mat1* locus in the chromosome is replicated only in one direction. Chromosomal inversion of the *mat1 ‘*cassette’ abolishes imprinting, which is partially restored by genetic manipulations promoting *mat1* replication in the opposite direction (7). Thus, replication of a specific *mat1* strand specifically by the lagging-strand replication complex is critical for generating the imprint. The imprinting process requires three genes: *swi1, swi3* and *swi7/pol*α (8–11). Mechanistically, Swi1 and Swi3 create a replication pause at the imprint site. They also block replication forks originating from the centromeric side of *mat1* (9). Furthermore, histone H3-Lys9 demethylases Lsd1/Lsd2 play a role in replication pause upstream of Swi1 and Swi3 (12). The single *swi7-1/pol*α imprinting-deficient mutant, however, shows a normal pause and normal replication fork block, and is, therefore, defective at some other undefined step in the imprinting pathway (9,11).

However, the nature of the imprint remains unresolved; it is thought to be either a site- and strand-specific nick (13) or an alkali-labile, RNase-sensitive modification, consisting of one or two ribonucleotides incorporated in the *mat1* DNA (14,15). The imprint creates a DNA fragile site, which is artifactually converted into double-strand break (DSB) due to hydrodynamic shear during DNA extraction from cells (7,13). Therefore, the imprint level is usually determined by quantifying the DSB at the *mat1* locus through Southern blot analysis.

The *swi7* gene, encoding the catalytic subunit of Polα, is inherently required in initiating both leading and lagging strand replication at the replication origins and for Okazaki fragment synthesis during the lagging-strand replication (11). The biochemical role of Polα/Swi7 in generating the imprint has remained elusive. Being an essential gene limits its analysis; only one allele, *swi7-1/pol*α (G1116E) in the catalytic subunit of Polα, is known to affect imprinting (11). Notably, the imprinting event occurs only on the newly synthesized lagging strand during S phase (16). Since the Polα/Primase complex can synthesize and extend an RNA moiety on DNA template, Polα/Swi7 is a plausible candidate for installing the imprint as a ribonucleotide(s) insertion at *mat1*, through the primase subunit. Alternatively, Polα-catalyzed DNA nicking may constitute the imprint (11). However, our results rule out the endonuclease function of Polα in generating the single strand nick. Instead, we show that even a catalytically dead Polα can install the ribonucleotide imprint. Furthermore, we identify Mcm10/Cdc23 as a new gene product required for installing the ribonucleotide imprint through its primase domain and interaction with Polα and Swi1.

## MATERIALS AND METHODS

### Strains and plasmids

The lists of strains, plasmids and oligos used in this study are provided in Supplementary Tables S1–S3, respectively (Supplementary Data). Media and growth conditions employed were as described (17). For viability assays, the cultures of the required strains were normalized to the same OD_600_, serially diluted 10-fold and 5μl of each dilution was spotted on the required plates. Plates were incubated at 25°C for 5 days or 30°C for 3–4 days and then photographed.

### Southern hybridization

**Figure 2.**
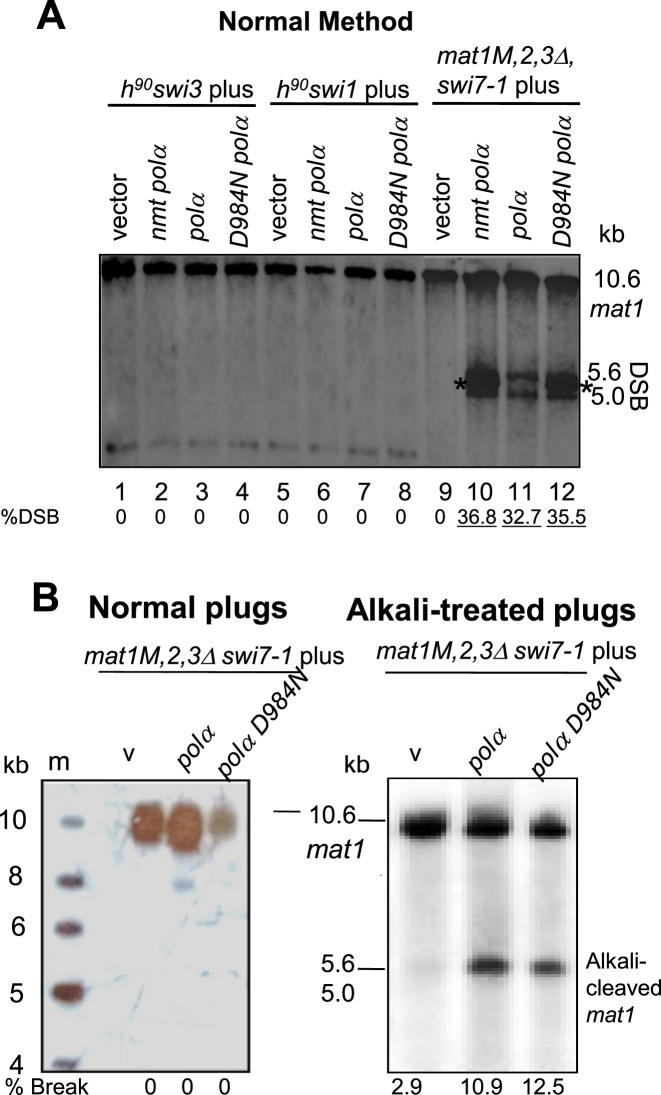
The catalytically dead *polα* (D984N) mutant complements the *swi7-1* imprinting defect. (**A**) Southern blot analysis. *polαD984N* mutant complements the imprinting defect of *swi7-1*, but not that of the *swi1* or *swi3* mutant, in donor-deleted strains. Indicated strains were transformed with vector, *nmtpolα* and *nmtpolαD984N* plasmids and growth media plates containing thiamine. Asterisk (*) indicates the cross hybridization with the high copy vector pREP3. (**B**) DNA was prepared from the donor deleted *swi7-1* mutant strains transformed with vector, *nmtpolα* and *nmtpolαD984N* plasmids and grown on selective plates containing thiamine. DNA was prepared in plugs (**B**) and digested with *Hin*dIII and Southern blotted followed by hybridization with radio-labelled *mat1M* probe (7). DNA was prepared in plugs and Southern blotted, without (left panel) or with alkali treatment (right panel), followed by hybridization.

DNA was prepared by the normal method as described earlier (17). DNA was isolated from yeast cells, digested with *Hin*dIII and subjected to agarose gel electrophoresis, followed by Southern blotting and hybridization, as described earlier [11]. Alternatively, DNA was prepared from cells embedded in agarose plugs and subjected to restriction endonuclease digestion and alkali treatment, as previously described (7). The 10.6kb *mat1M*–containing *Hin*dIII fragment was used as a probe for Southern hybridization (Figure 1A). For experiments shown in Figure 2, donor deleted strain was used containing only the *mat1* locus, with *mat2* and *mat3* loci being deleted (18). Normally, the level of DSB ranges around 25% of the *mat1* DNA when DNA is prepared by the conventional method. However, we often observe a higher level of ∼35% because of partial shearing of the *mat1* DNA. Furthermore, the level of DSB observed in alkali blots is ∼10–12 % of *mat1* DNA. The 2–3-fold difference between the two methods of DNA preparation can be explained by the fact that blots of alkali-treated plugs detect only the fragments of the imprinted strand while in case of DNA prepared by the normal method both strands are detected as the *mat1* DNA undergoes a DSB.

### Quantitation of switching efficiency

The homothallic (*h^90^*) cells, efficiently switch their mating type. Thereby, yeast colonies are composed of an equal proportion of P and M cells. Cells of opposite type mate under nitrogen starvation conditions and the resulting ‘zygotic’ diploid cell undergoes meiosis and sporulation to produce four haploid spores, called ascospores. The per cent switching efficiency was determined by using the equation: 100 × [{2(number of zygotes)}/{2(umber of zygotes) + number of vegetative cells}].

Also, the spores synthesize starch but the vegetative growing cells do not. Because starch readily reacts with iodine vapors, colonies of efficiently switching wild type strain stain black in colour while those of switching defective mutants stain lighter when exposed to iodine vapors (8,17). This procedure was used to test complementation of switching defective mutants. The transformant strains containing plasmid borne *polα* or *cdc23* genes, expressed under the control of *nmt1* or *nmt41* promoters, were assayed for complementation on plates containing the repressor, thiamine. Under these conditions, leaky expression was observed from the *nmt1* promoter (19; Ahmed and Singh, unpublished).

Quantitation of DSB as a measure of switching was done by using the formula: [A/(A + B)] × 100, where A represents the combined band intensity of 5.6 and 5.0 kb bands generated by DSB and B, the 10.6 kb band of the *mat1* locus.

### Pull-down experiments

Vector constructs encoding MBP-tagged *polα* or MBP-tagged *swi7-1*/*polα* gene, cloned in the expression vector pMALp2 were expressed by inducing with 1mM IPTG for 8hrs at 25°C. MBP-Polα or MBP-Polα^Swi7-1^were purified using amylose resin (NEB) according to manufacturer's instructions.

Genes encoding *cdc23* and *cdc23M36* were cloned into GST fusion vector pGEX2T and were expressed in Codon Plus strain by inducing with 1mM IPTG for 16 hrs at 18°C.For the pulldown assay, 2mg of MBP-Polα^+^ or MBP-Polα^Swi7-1^ extracts were first bound to the amylose resin and then extracts containing ultrafilter-concentrated GST-tagged Cdc23 or Cdc23-M36 were added at increasing concentration from 1 to 4 mg. After incubation at 4°C for 4 h, the amylose resin was washed with 10 column volumes of binding buffer (20mM Tris pH 7.4, 10 mM EDTA and 2 mM β-mercaptoethanol), boiled in 50 μl of SDS loading buffer for 5min and subjected to western blot analysis by using antibodies against MBP and GST at 1:1000 dilution.

Western blots were visualized by using the ECL kit (GE Healthcare).

### Co-immunoprecipitation experiments

Cells of strains having the genotype *cdc23*-HA/*swi1*-TAP and *cdc23*HA/*swi3*-myc as well as untagged strains were harvested by centrifugation and washed once with ice-cold lysis buffer (150 mM NaCl, 50 mM Tris–HCl pH 7.5, 50 mM NaF, 5 mM EDTA, 0.1% PMSF) containing 60 mM β-glycerophosphate. An equal volume of Zirconium beads (Sigma) was added and cells were broken by shaking in a mixer mill (Bead Beater) for 15 min at 4°C, intermittently for an hour. Zirconium beads and cell debris were removed by centrifugation for 30–35 min followed by centrifugation for another 10 min in a microfuge at 4°C.

For analysis of whole-cell extracts, 2 × Läemmeli sample buffer was added to the supernatant and the mix was boiled for 5 min at 94°C. For immunoprecipitations, extracts containing ∼1–1.5 mg of total protein were mixed with either α-CBP (Calmodulin Binding Protein; Millipore Sigma, 1μg/ml lysate), α-HA (Santacruz, 1 μg/ml lysate) or α-Myc (SantaCruz, 1.5 μg/ml lysate) specific antibodies. After overnight incubation at 4°C, 50 μl of protein A–agarose was added to total 1–1.5 mg of protein lysate and the mixtures incubated for another 4 h at 4°C. Beads were collected by centrifugation at 1000 rpm, washed three times with lysis buffer, resuspended in Läemmli sample buffer and boiled for 10 min. Western analyses were performed using Mouse monoclonal antibodies anti-HA (1:2000), anti-Myc (1:2500), and anti-CBP (1:2500) for detection of HA- tagged Cdc23 (from cells overexpressing *cdc23* gene cloned in the vector pREP41HAN), myc-tagged Swi3 and TAP-tagged Swi1, respectively. For inputs, 100 μg protein (∼7.5–10%) was loaded on the SDS-PAGE before the transfer with 5–10% methanol and Tris–Cl buffer. Polyclonal antibody was raised against purified MBP-tagged Polα in rabbits and precleared by binding to MBP-Amylose resin before using for western blotting at dilution of 1:2000.

### Data aquisition and analysis

The images were scanned for all the blots using the digital systems for enhanced chemiluminescence and autoradiograph, using the Biorad Pharos FX Plus Molecular Imager. The linear range was determined through the system by detection of saturation. The exposures for westerns were kept constant for each blot. The image quantitation was performed using Image J software. Equal concentrations of proteins were used and normalized with an internal control. Replicates and repeats were performed to ensure the data did not suffer experimental bias. The statistical analysis was done using one-way ANOVA followed by Tukey's multiple comparisons. Multiple comparisons with pairwise comparisons was performed within all columns using Graphed Prism software. For autorads, the automatic detection system during scanning, minimized the background and saturation. The levels of DNA and protein were constant in each experiment. The statistical analysis was done using one-way ANOVA followed by Tukey's multiple comparisons. Multiple comparisons with pairwise comparisons were performed within all columns using Graphpad Prism software.’

### Directionality assay

The preferential mating type displayed by a strain was determined by PCR according to ref. 20). The reaction included 25ng of genomic DNA and primers, which included the common primer MT1, *mat1P*-specific primer MP and *mat1M*-specific primer MM; Table S3), which yielded *mat1P* specific product of 987 bp and *mat1M*-specific product of 729 bp. The extent of directionality was determined by calculating the ratio of signals of the Plus/Minus mating type, which is close to 1 in wt h^90^ strain and deviates significantly from 1 in the mutants displaying the directionality defect.

#### Far-western blotting

Far-western blotting was carried out as reported (21).

## RESULTS

### Polα catalytic subunit does not play a direct role in imprinting

It was reported earlier that the DNA prepared by the normal method causes hydrodynamic shearing, resulting in conversion of a nick into double strand break at the imprint site (13). As a result the *mat1 Hin*dIII fragment of 10.6kb is split into two bands of 5.6 and 5.0 kb (Figure 1B, C). According to this study, preparation of the DNA in agarose plugs avoids the hydrodynamic shear and *mat1* DNA shows a nick in the top DNA strand as the putative imprint (13). Therefore, we first investigated whether the catalytic subunit of Polα may be directly involved in generating the imprint in the form of a nick at the *mat1* locus. One family of homing endonucleases contains the LAGLIDADG motif (4). Interestingly, a region in Polα of *S. pombe* shares similarity with the LAGLIDADG motif (22–24; Supplementary Figure S1A). To ascertain the role of this motif in imprinting, both of the conserved Asp (D) residues at positions 1158 and 1160 labeled D1 and D2 (Supplementary Figure S1A) in the *polα* gene were individually substituted with Ala (A) residue. After cloning them into the pART1 vector containing the constitutive *adh1* promoter, both wt and mutated genes were integrated into the resident *swi7-1* allele by homologous recombination (Supplementary Figure S1B). Increased iodine staining of the transformant colonies showed that, like intact episomal *polα*, both wt and *polα* D to A single and double mutant constructs restored switching when chromosomally integrated by homologous recombination into the *swi7-1* mutant (Supplementary Figure S1C). Thus, the putative DADG region is not involved in imprinting.

Polα also contains the restriction endonuclease (REase) signature sequence called PD-(D/E)XK (25) within the region-V (Supplementary Figure S1D). The region spanning the residues 1040 to 1056 with the sequence LDSQGKPNLDVKGLDMK contains two overlapping LD-(D/E)XK motifs: LD—X_(7)_—DVK and LD—X_(12)_—DMK. The critical Asp (D) residues are located at positions 1041 and 1049 and at residues 1041 and 1054, respectively (Supplementary Figure S1D). Each of the Asp residues at positions 1041, 1049 and 1054 was mutated to Ala. After cloning into pART1, the DNA of wt and mutant *polα* was transformed into *swi7-1* mutant after linearization at *Bgl*II site. However, like the wt *polα* gene, all three mutants restored iodine staining/switching (Supplementary Figure S1E), ruling out the role of PD(D/E)XK motif in imprinting.

We further tested whether Polα exhibits a *mat1*-specific endonuclease activity. We observed that the largely supercoiled (sc) form of Bluescript vector containing the *mat1M* fragment was efficiently converted into open circular form (oc) upon incubation with MBP-Polα but not so effectively by MBP alone (Supplementary Data; Supplementary Figure S2A). No effect was observed upon incubation of the linearized *mat1M* plasmid DNA (Supplementary Figure S2B). However, sequencing of the purified oc DNA obtained upon incubation with MBP or MBP-Polα did not show any discontinuity or decrease in the sequencing signal coinciding with the known site of the imprint (inverted arrows; Supplementary Figure S2C, D). Thus, Polα may only be associated with a random endonuclease activity, which acts on supercoiled DNA but not specifically on *mat1* DNA.

Next, we asked whether the integrity of Polα in the replication complex or its catalytic activity of Okazaki fragment synthesis establishes the imprint. Mutation of the conserved Asp residue in ‘region-I’ (the most conserved region of the α-like DNA polymerases) of human *polα* to Asn (D1004N) is known to abolish the catalytic activity without altering the structure or stability of the Polα–primase complex (26,27). The corresponding Asp residue (D984) in the fission yeast Pol*α* exerts a dominant negative growth defect on cells similar to that of the human Polα D1004N mutation (26,27). We checked whether the *polα* D984N mutation affects imprinting. Transformants were plated on the thiamine repressor-containing medium to minimize the deleterious effect of the (D984N) mutation. Under these conditions, the wild type *polα* gene complemented the *swi7-1* mutation (Ahmed and Singh, unpublished), likely due to leaky expression of the *nmt1* promoter. To avoid the complication of donor loci alterations, and because *mat1* locus is normally imprinted in the *mat2* and *mat3* donors-deleted cells (18), we also tested the effect of *polα* (D984N) on imprinting in the donors-deleted *swi7-1* mutant by transforming it with either vector control, *polα* or *polα* (D984N) plasmid. The transformants were grown in the presence of the repressor thiamine. Southern blot analysis showed that the DSB level is restored in the donor-deleted *swi7-1* cells by the D984N plasmid (Figure 2A, lanes 9–12) but not in *swi3-157* or *swi1-111* mutant (Figure 2A, lanes 1–8). Thus, the *pol*α (D984N) mutation only complemented the imprinting defect of the *swi7-1* mutant and not that of the other two known *swi1* and *swi3* imprinting-deficient mutants. These results also suggest the requirement of Swi1 and Swi3 for the complementation by *polα D984N* mutant.

Results so far failed to identify any endonuclease like function in Polα. Although this does not completely rule out the role of a Polα-associated nuclease, we also considered it unlikely as broken DNA molecules are subject to the action of nucleases and lead to lethality. Although appearance of break in DNA prepared in alkali-treated plugs does not distinguish between the presence of a nick or a ribonucleotide insertion, we noted that Dalgaard and Klar failed to detect a nick at *mat1* locus when genomic DNA of wt cells was prepared in agarose plugs and subjected to denaturing gel electrophoresis in the presence of formaldehyde (7).

These considerations led us to the possibility of the existence of a ribonucleotide bond at the imprint site. This possibility was tested by performing the experiment according to Dalgaard and Klar (7), by preparing the DNA of the transformants of the *swi7-1* mutant with *polα* and *polα D984N* in agarose plugs, followed by *Hin*dIII digestion and electrophoresis with and without treatment of plugs with alkali, Southern blotting and hybridization, as done earlier. The results show a lack of any band at 5–5.6 kb in DNA of *swi7-1* mutant transformed with vector, *polα* and *polα* D984N plasmids prepared in plugs (Figure 2B, left panel). Surprisingly, a broad band was observed at 5–5.6kb when the DNA of cells transformed with wt *polα* as well as *polα D984N* plasmid was prepared in plugs and subjected to alkali treatment before electrophoresis (Figure 2B, right panel). These results led us to the conclusion that both *polα* and *polαD984N* generate an alkali-labile bond. Thus, the imprint is likely to be constituted by a mono- or di-ribonucleotide insertion in the *mat1* DNA, as reported (7,15).

**Figure 3.**
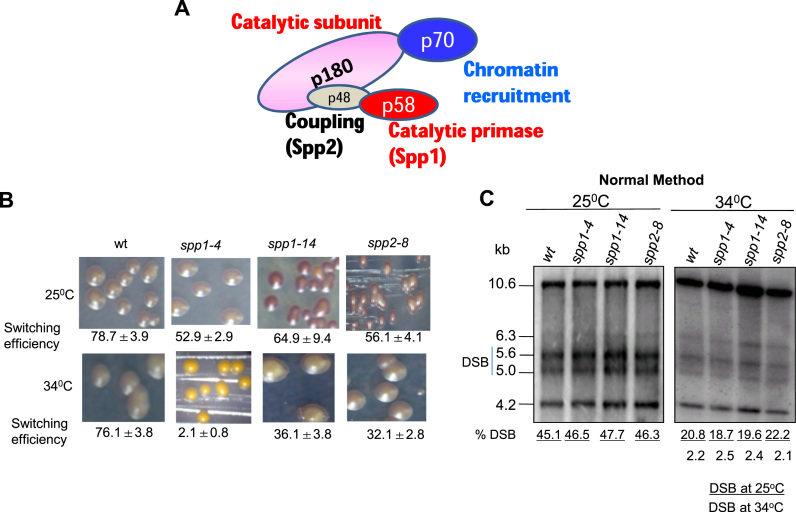
Polα subunit s*pp1* and *spp2* mutants are not defective in imprinting. (**A**) Sub unit structure of DNA Polα shows the largest catalytic sub unit (p180), primase sub unit Spp1 (p59), coupling subunit Spp2 (p48) and the p70 sub unit required for chromatin recruitment. (**B**) Picture of the iodine-staining phenotype of colonies of strains grown/sporulated at the indicated temperatures. Numbers denote the efficiency of switching represented as percent level of zygotic asci. (**C**) Southern blot analysis of strains described in (**B**). DNA was prepared by the normal method. Numbers indicate the level of DSB.

These results created a conundrum: How might the catalytically dead Polα complement the imprinting defect of *swi-1* mutant? The *polα^D984N^* mutation in the evolutionarily conserved Asp residue does not alter either the stability or assembly of the mutant Polα–primase complex (26). Notably, the catalytic subunit mutant protein is unable to further extend RNA primers synthesized by the primase subunit (26). We envisaged two possibilities: either the Polα^Swi7-1^ mutant protein complex is defective in the primase activity or in utilizing the RNA primer synthesized by the primase subunit for DNA synthesis. To distinguish between these possibilities, we tested the imprinting and switching efficiency of temperature sensitive mutants in subunits of Polα: *spp1-4, spp1-14* alleles of the primase *spp1* and *spp2-8* allele of *spp2* gene (Figure 3A; 29). Although, somewhat reduced efficiency of switching was observed, especially in *spp1-4* mutant (Figure 3B), the level of imprint/DSB was affected in these mutants to an extent similar to the wt strain when cultured at 34°C (Figure 3C). Thus, these results do not support a role of the Polα-primase subunit in *mat1* imprinting. However, the extremely low rate of switching in the *spp1-4* mutant at 34°C may be due to lower efficiency of utilization of the imprint for switching, as observed earlier in case of *swi2, swi5* and *swi6* mutants (8).

Interestingly, we find that, similar to *swi6* mutant, the *spp1-4* mutant shows a defect in directionality of switching; it contains nearly 20 fold more *mat1M* than *mat1P* DNA (Supplementary Data; Supplementary Figure S3A, B), indicating that like *swi6*, the *spp1-4* mutant displays a defect in directionality of switching from Minus to Plus mating type.

### Cdc23/SpMcm10 performs the imprinting function at *mat1*

**Figure 4.**
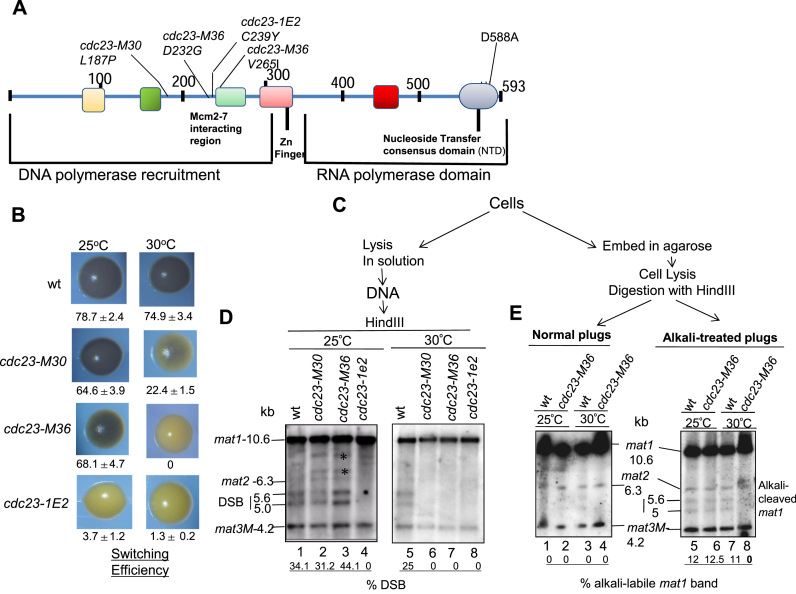
Role of Cdc23/SpMcm10 in Imprinting at the *mat1* locus. (**A**) Structure of Cdc23 protein, indicating the N-terminal domain required for binding with ssDNA and DNA Polα, the Zinc finger domain and the C-terminal domain performing the RNA polymerase function. Locations of mutations *M30, M36* and *1E2* in the N-terminal domain and D588A in the C-terminal RNA polymerase domain are shown (adapted from ref. 30). (**B**) Iodine-staining phenotypes of the indicated strains in *h^90^* background after growth on PMA^+^ plates at 25°C or 30°C. Numbers indicate switching efficiency, as represented by level of zygotic asci and vegetative cells. Experiment could not be performed with the *cdc23-D588A* mutant due to its lack of viability (ref. 30). (**C**) Schematic showing the process of preparation of DNA by normal method or in plugs for experiments shown in (**D**) and (**E**). *represents bands due to mating type rearrangements. (**D**) *cdc23* mutants exhibit imprinting defect. DNA was prepared and analyzed by the standard method. (**E**) DNA was prepared in plugs from indicated cultures grown at 25°C or 30°C, without (left panel) and with alkali treatment (right panel) followed by Southern blotting and hybridization. Numbers represent the level of imprint in the *mat1* DNA.

Because of a reported primase-like function of Mcm10/Cdc23, we next investigated the efficiency of switching and *mat1* imprinting of *cdc23* mutants (Figure 4A). Previous structure-function analysis of Cdc23 has revealed three functional domains: Polα- interaction domain, zinc domain and putative primase domain, having similarity to the bacteriophage T7 gene 4 primase (Figure 4A) (30). Notably, the *cdc23-M30, cdc23-M36* and *cdc23-1E2* mutations are located in its Mcm/Polα-interacting domain (30). Both *cdc23-M30* and *cdc23-M36* mutants showed normal iodine staining and switching (Figure 4B, left panel) at permissive temperature (25°C) but reduced iodine staining and switching at 30°C semi-permissive growth temperature (Figure 4B, right panel). In contrast, the *cdc23-1E2* mutant (Figure 4A), showed low iodine staining and switching efficiency at both growth temperatures (Figure 4B). The effect on iodine staining was paralleled by the effect on level of haploid zygotic asci confirming that the low iodine staining was due to reduced switching (Supplementary Figure S4).

The effect on imprinting was assessed by determining the effect of temperature on the level of DSB; for ease we prepared the DNA by conventional method. Results show that both *cdc23-30* and *cdc23-36* mutants contained normal level of DSB at 25°C but reduced DSB at 30°C (Figure 4D, compare lanes 2 and 3 with lanes 6 and 7), while *cdc23-1e2* mutant showed reduced DSB at both the temperatures, paralleling the effect of growth temperature on switching (Figure 4D, compare lanes 4 and 8). Importantly, results of experiments done with DNA prepared in plugs and subjected to electrophoresis with and without alkali treatment showed that, as indicated by the bands of 5.6 and 5.0 kb, the *cdc23-M36* mutant contains an alkali-labile bond at *mat1* when grown at 25°C but not at 30°C (Figure 4E, right panel; compare lanes 6 and 8). Thus, Mcm- and Polα-interacting domain of Cdc23 plays a role in installing the alkali-labile imprint.

**Figure 5.**
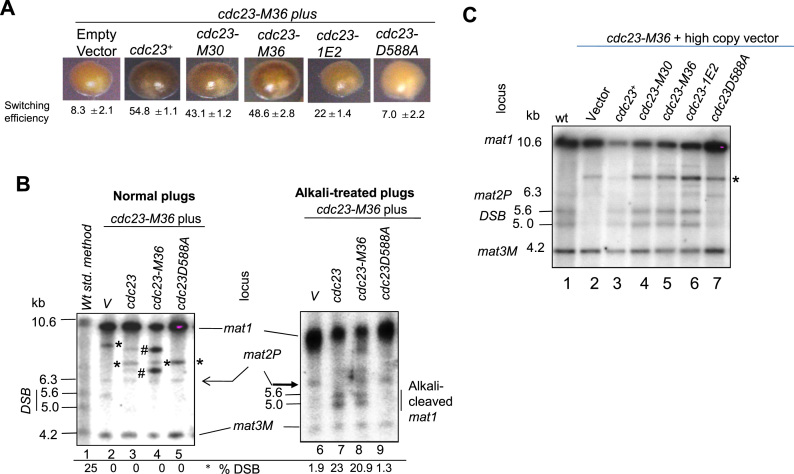
Primase domain of Cdc23 is essential for imprinting. (**A**) Iodine-staining phenotypes of *h^90^, cdc23-M36* mutant transformed with plasmids bearing indicated *cdc23* gene mutations and grown on selective medium plates at 30°C. (**B**, **C**) *cdc23* genes having mutation in primase domain fail to complement the imprinting defect of the *cdc23-M36* mutant. DNA was prepared from transformants of *cdc23-M36* mutant with the high copy plasmids containing the indicated genes and grown at 30°C. (**B**) DNA was prepared in agarose plugs. Lane labeled wt indicates DNA prepared from wild type strain. Vector only lane shows cross-reacting vectors backbone bands (marked by *) present in all lanes, while bands marked (#) correspond to mating-type rearrangements. DNA prepared in plugs from strains grown at 30°C was digested with *Hin*dIII, followed by agarose gel electrophoresis without (left panel) and with alkali treatment (right panel). (**C**) DNA prepared from the indicated transformant cells grown at 30°C by the normal method was digested with *Hin*dIII followed by agarose gel relectrophoresis. After Southern blotting membranes were subjected to hybridization. * represents cross reacting band of the vector DNA.

Because the primase mutant is inviable (30), we explored the role of Cdc23 domains in imprinting by genetic complementation experiments. We tested the ability of the high copy number plasmids bearing different *cdc23* mutant alleles to suppress the imprinting defect of the *cdc23-M36* allele. While plasmids bearing wild-type *cdc23^+^, cdc23-M30, cdc23-M3*6 or *cdc23-1E2* complemented the switching defect of the *cdc23-M36* mutant grown at 30°C, the primase defective mutant c*dc23-D588A* (30) did not (Figure 5A). Results with DNA prepared in plugs with and without alkali treatment showed that plasmids bearing *cdc23^+^* and *cdc23-M36* restored the alkali-labile bond at *mat1* in *cdc23-M36* mutants grown at 30°C (Figure 5B, compare lanes 7 and 8 with lanes 3 and 4 ), while the primase defective *cdc23-D588A* gene did not (Figure 5B, lane 9). Similar results were obtained when DNA prepared from the transformants by the normal method was subjected to Southern blot hybridization (Figure 5C). Surprisingly, all three *cdc23* mutants in the Polα-interacting domain-*M30, M36* and *1E2* complement the imprinting defect of *cdc23-m36* mutant at 30°C (Figure 5C, lanes 4–6). Importantly, the mutant D588A did not restore the imprint/DSB in the *cdc23-M36* mutant (Figure 5C, lane 7). It is possible that the effect of *cdc23-D588A* mutant gene on *cdc23-M36* mutant may be due to a dominant negative effect on imprinting and switching. However, this possibility was ruled out as the D588A mutant gene had no effect on switching in the wt *h^90^* strain (Supplementary Figure S5).

### Genetic and biochemical interactions of Cdc23 with Swi1, Swi3 and Swi7

Polα physically interacts with Cdc23 (30–33). Since both *cdc23* and *swi7-1*/*polα* mutants are defective in imprinting, we tested genetic interaction between their mutations. The double mutant showed much reduced switching efficiency and sporulation on minimal medium at 30°C (Supplementary Data, Supplementary Figure S6A). This result indicates that both these factors are required at the same step in imprinting. Surprisingly, unlike the single mutants, the double mutant failed to grow on rich media at 30°C (Supplementary Data, Supplementary Figure S6C).

We also investigated genetic interactions of *cdc23* mutant with *swi1* and *swi3* mutants in switching and viability. Interestingly, *cdc23-M36* mutant showed cumulative effect on switching efficiency in combination with *swi1* and *swi3* mutants on minimal medium at 30°C (Supplementary Figure S6B). The double mutants of *cdc23* with *swi1* and *swi3* mutants also showed synthetic lethality on rich medium at 30°C (Supplementary Figure S6D). The discrepancy between growth on minimal medium and lack of growth on rich medium at 30°C is surprising. It may reflect that the double mutants of *cdc23M36* with *swi1, swi3* and *swi7* may affect some important physiological function during vegetative growth but not during starvation.

Interestingly, co-immunoprecipitation experiments also showed that Cdc23 interacts with Swi1 but not Swi3 *in vivo* (Supplementary Data, Supplementary Figure S7A, B). Thus, Cdc23 may also act at a common step involving Swi1 and Polα.

**Figure 6.**
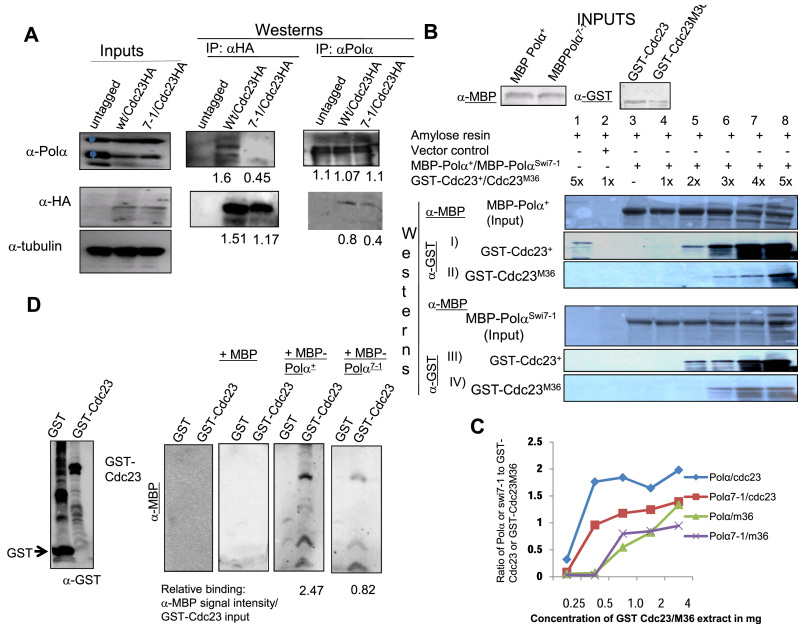
Reduced interaction of Cdc23 and Polα mutant proteins. (**A**) Co-IP of HA-tagged Cdc23 with Polα and Polα^swi7-1^. Inputs blot shows equivalent amounts of HA-tagged Cdc23, Polα and Polα^swi7-1^ was present in the indicated strains. Polα appears as a doublet, as indicated by arrowheads. IP was performed with anti-HA antibody followed by immunoblotting with anti-HA and anti-Polα antibodies. (B–D) *In vitro* interactions. (B, C) Pull-down assay. (**B**) Identical amounts of MBP-Polα (panels I and II) and MBP-Polα^swi7-1^ (panels III and IV) were bound to amylose beads and incubated with increasing concentrations of the normalized amounts of GST-Cdc23 (panels I and III) and GST-Cdc23^M36^ (panels II and IV), as indicated. Following SDS-PAGE of the bound proteins, the blots were probed with anti-MBP (panels I and II) and anti-GST antibodies (panels III and IV). (**C**) Quantitation of MBP-Polα and MPB-Polα^swi7-1^ binding with Cdc23 and Cdc23^M36^. X axis shows the concentration of Cdc23 or Cdc23^M36^ and the Y axis shows the ratio of Polα or Polα^swi7-1^ to input GST-Cdc23 or GST-Cdc23^M36^. (**D**) Far-western analysis. Partially purified GST and GST-tagged Cdc23 were subjected to SDS-PAGE and western blotted. The blots were subjected to progressive renaturing conditions, purified extracts from *E. coli* strains expressing MBP, MBP-Polα^+^ and MBP-Polα^Swi7-1^, as indicated. Blots were probed with anti-Polα (1:1000), anti-HA, anti-GST and anti α-tubulin (1:2,000) antibodies. Control indicates extract from *E. coli* control strain.

We further investigated direct biochemical interactions *in vivo* and *in vitro*. Polα^+^p could be readily co-immunoprecipitated with Cdc23^+^-HA but in comparison the mutant Polα^7-1^p was less efficiently co-immunoprecipitated (Figure 6A; arrowheads). *In vitro* pull-down experiments showed that MBP-Polα^+^ interacted more strongly with GST-Cdc23 than with GST-Cdc23^M36^ (Figure 6B, panels I and II; Figure 6C). Likewise, Polα^7-1^ interacted more strongly with Cdc23^+^ than with Cdc23^M36^ (Figure 6B, panels III and IV). Furthermore, Cdc23 interacted with Polα^+^ more strongly than with Polα^7-1^ (Figure 6B, compare panels I and III, panels II and IV; Figure 6C). The order of strength of interaction was Polα^+^-Cdc23^+^ > Polα^Swi7-1^-Cdc23^+^ > Polα^+^- Cdc23-M36 = Polα^Swi7-1^-Cdc23-M36. As Cdc23 recruits Polα to chromatin (34–36), our results suggest that the recruitment of Polα may be reduced in *swi7-1* and *cdc23-M36* mutants. Far-western analysis showed nearly 3-fold weaker interaction of Cdc23 with Polα^swi7-1^ as compared to Polα^+^ (Figure 6D).

A trivial possibility to explain our genetic results could be reduction in the level of Polα in *cdc23* mutants, as inactivation of Cdc23 homolog, Mcm10, is known to cause rapid loss of Polα in *S. cerevisiae* (34–36). However, western blot analysis shows that, compared to the wild type strain, the level of Polα is not reduced significantly in *cdc23-M36* mutant grown at 25°C and 30°C (Supplementary Figure S8A, B).

### MCM helicase subunits do not play a role in imprinting

Our findings indicated a role of DNA replication initiation in imprinting. Next, we queried whether the components of MCM-helicase complex, essential for DNA replication initiation, are required for imprinting (Supplementary Figure S9A). We observed normal iodine staining of colonies of mutants in *mcm2* (*cdc19-p1*) (37), *mcm4* (*cdc21-M68*) (38), *mcm5* (*nda4-108*) (39) and *mcm6* (*mis5-268*) (40), in homothallic (*h^90^*) background, indicative of normal switching at permissive temperature (25°C) and semi-permissive temperature (30°C) (Supplementary Data; Supplementary Figure S9B). This result argues against the role of earlier steps of replication initiation in imprinting.

## DISCUSSION

This study addressed the mechanism of action of the catalytic subunit of Polα in strand-specific imprinting at the *mat1* locus in *S. pombe*. While Swi1 and Swi3 play an indirect role by providing a paused replication fork, the enzymatic activity catalyzing the imprint is not known. Given its role in lagging strand synthesis (11,41), we investigated whether the catalytic subunit of Polα plays a direct role in generating a nick or ribonucleotide insertion. Results showing lack of a role of endonuclease motifs in imprinting as well as lack of a specific endonuclease activity of recombinant Polα argues against a nick being generated by Polα at the *mat1* locus. Most surprisingly, even a catalytically dead *polα*^D984N^ mutant gene was complementation proficient. Likewise, the *spp1* and *spp2* subunit mutants showed normal imprinting. None of the components of the MCM helicase complex was found to be involved in imprinting. Finally, mutants of the non-canonical primase *mcm10*/*cdc23* gene were found to be defective in imprinting. These results support the ribonucleotide nature of the imprint. We show further that Cdc23 inserts the imprint through its primase domain and interaction with Swi1 and DNA Polα.

Mcm10/Cdc23 has been studied in *S. cerevisiae, S. pombe* and metazoans. However, primase activity has been demonstrated in detail in *S. pombe* but not in the vertebrate orthologs (30–32,42). *In vitro* studies have shown that the SpCdc23 makes 2–20nt long RNA primers, which are then transferred to and extended by the catalytic subunit of Polα (41). It is pertinent that even a short, 2-ribonucleotide primer synthesized by Cdc23 can be extended into a DNA chain by Polα (30,32). We suggest that primers as short as two ribonucleotides, that are synthesized by primase domain of Cdc23 at the *mat1* locus, can be extended into a DNA chain by the catalytic domain of Polα; Polα is recruited through N-terminal domain of Cdc23 (4). It is puzzling how such an event could occur at the pause site. In this regard it has been shown that multiple rounds of primer synthesis occur at replication pause site (7,43). Furthermore, these two ribonucleotides may not be processed by RNaseH and could be ligated with the 3′-end of the adjacent Okazaki fragment, thus establishing the imprint.

Furthermore, the reduced binding of Cdc23-M36 to Polα^+^ and still poorer binding to Polα^7-1^, may impede extension of RNA primers by the Polα^7-1^. This idea can explain the cumulative reduction in imprinting/switching of *cdc23-M36, polα^7-1^* double mutant on synthetic medium at 30°C. The ability of Polα^D984N^ to restore imprinting in *swi7-1* mutant may be ascribed to possible restoration of recruitment of Polα^swi7-1^ to a Cdc23-bound template to help extend the RNA primers. The interaction of Cdc23 with Swi1 also suggests that the unique primase activity may occur through interaction of Cdc23 with Polα and Swi1 at the pause site.

Another puzzling observation is the ability of the *cdc23* mutant genes *M30, M36* and *1E2* to complement the imprinting defect of the *cdc23-M36* mutant. This can be explained by envisaging the dosage effect wherein extra amount of the above mutant proteins may compensate for the weaker interaction of the mutant protein with Polα. The inability of similarly overexpressed *cdc23-D588A* mutant gene to complement the imprinting defect of *cdc23-M36* mutant also rules out an indirect effect and lends support to the role of primase domain of Cdc23 in inserting the ribonucleotide imprint.

**Figure 7.**
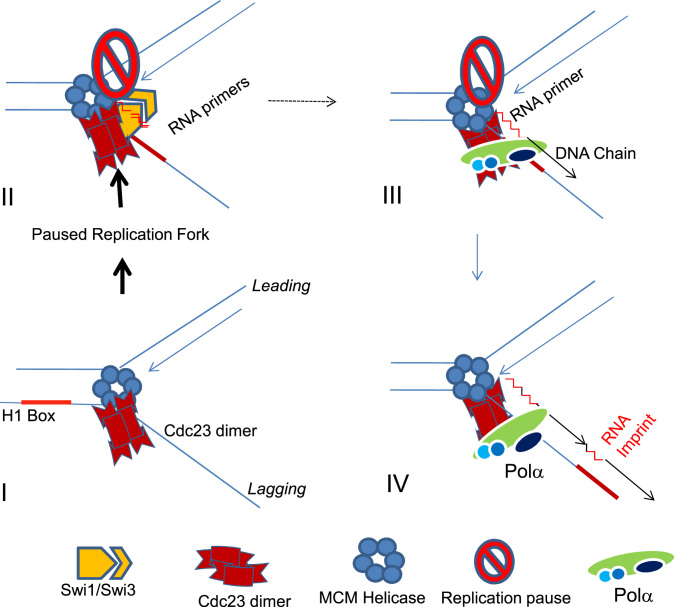
A model visualizing the role of Cdc23 Primase and DNA Polα during lagging strand synthesis in generating the imprint at the pause site at the junction of the H1 box and allele-specific sequence at the *mat1* locus. Pausing at the imprint site by Swi1 and Swi3 may lead to allow Cdc23 primase to interact with Swi1 and to linger at the pause site for prolonged period during elongation phase of DNA replication (II). This may allow synthesis of a short primer of two ribonucleotides length (II), which is subsequently extended by the catalytic subunit of Polα (III). The imprint may persist due to ligation with the next adjoining Okazaki fragment synthesized further upstream (IV).

How imprinting is caused uniquely at the *mat1* locus remains a puzzle. One possibility is that nucleotide sequence and unique DNA/nucleoprotein architecture at the *mat1* locus leads to a Swi1- and Swi3-dependent pause at the imprint site. This pause may facilitate localization of Polα to persist at the imprint site long enough to synthesize a 2-ribonucleotide primer, which is then extended by the catalytic subunit of Polα during the elongation stage of DNA replication (Figure 7). A weaker interaction of mutant Cdc23 with Polα protein may also cause reduced sequence specificity and/or efficiency to installing the imprint. Indeed, multiple break sites have been reported distal to the imprint site at the *mat1* locus in the *swi7-1* mutant (44). Again, possibly due to weaker interaction with Cdc23, the mutant Polα^Swi7-1^ may extend multiple ribonucleotide insertions near the imprint site. In sum, our results suggest that Cdc23 plays a more direct role in imprinting than the previously described factors.

Interestingly, the residue D588 is conserved between *S. pombe* and metazonas, including human, mice, Xenopus (30), though the metazoan homologs have been shown to lack primase activity *in vitro* (31). However, given the presence of an extra domain in the metazoan orthologs (30,42), a developmental control of primase activity through the extra domain may occur. Further studies are required to investigate such a possibility.

The idea of strand-specific incorporation of ribonucleotides as a part of asymmetric DNA replication contributing to generation of developmental asymmetry of sister cells appears to be unique (4). Indeed a similar mechanism was shown recently in the evolutionarily distant yeast, *Schizosaccharomyces japonicus* (45).

Widespread incorporation of ribonucleotides has indeed been reported during mitochondrial DNA replication (46) and replication by both Polα and Polδ *in vitro* (47). Thus, ribonucleotide insertion in DNA may play a role during differentiation or to facilitate recombination (48). Similarly, fragile sites occur in mammalian genome predominantly in AT-rich sequences with a potential to form stem-loop structures in response to the inhibitors of DNA replication by Polα (49). Interestingly, a homozygous mutation in Mcm10 in mice causes defect during morula to blastocyst stage, the stage when the inner cell mass (ICM) is formed, leading to formation of different organs (50). Thus, it would be interesting to investigate the role of Mcm10 in generation of DNA fragile sites, the incorporation of ribonucleotide insertions at these sites and their possible role in asymmetric cell differentiation during development in metazoans.

## Supplementary Material

Supplementary Data
