# Supplementary material for: Role of Cdc23/Mcm10 in generating the ribonucleotide imprint at the mat1 locus in fission yeast
Source: Nucleic Acids Res. 2019 Feb 13;47(7):3422–33. doi: 10.1093/nar/gkz092 (PMC6468313; doi:10.1093/nar/gkz092)
Supplement: Supplementary Data [file gkz092_supplemental_file.pdf]

Supplementary Data

Table S1. List of strains used in the study

Strain Name	Genotype	Source
SA220	*h^{+}, cdc19-P1, leu1-32, ade6-M216, ura4D18*	S. Kearsey
SA224	*h^{+}, mis5-268, leu1-32, ade6-M216, ura4D18*	S. Kearsey
SA226	*h^{+}, nda4-108, leu1-32, ade6-M216, ura4D18*	S. Kearsey
917	*h^{-}, cdc23-M30*	S. Kearsey
893	*h^{-}, cdc23-1E2, leu1-32*	S. Kearsey
155	*h^{-}, cdc23-M36*	P. Nurse
HE9	*h^{90}, swi1-1*	This study
SPJ802	*h^{90}, leu1-32, ura4D18, spp1-4, ade6-M216*	This study
SPJ808	*h^{90}, leu1-32, ura4D18, ade6-M210, spp1-14*	This study
SPJ811	*h^{90}, leu1-32, his2, ura4D18, ade6-M216, spp2-8*	This study
KSP85	*h^{90}, leu1-32, his2, ade6-M210, swi1-1*	This study
Bsp201	*h^{90}, leu1-32, uraD18, ade6-M216, his2, swi1-111*	This study
Bsp332	*h^{90}, leu1-32, ura4D18, ade6-M216, swi1-111, cdc23 M36*	This study
SP808	*mat1M, mat2,3Δ::LEU2, ade6-M210, leu1-32*	This study
KSP59	*mat1M, mat2,3Δ::LEU2, leu1-32, ade6-M216, swi7-1*	This study
Bsp142	*h^{90}, leu1-32, ura4D18, ade6-M216, his2, cdc23-M36*	This study
Bsp135	*h^{90}, leu1-32, ura4D18, ade6-M210, his2 , cdc23-M36, swi7-1*	This study
Bsp124	*h^{90}, leu1-32, ura4D18, ade6-M216, swi7-1, cdc23HA::LEU2*	This study
Bsp126	*h^{90}, leu1-32, ura4D18, ade6-M216, cdc23HA::LEU2*	This study
Bsp128	*h^{90}, leu1-32, ura4D18, ade6-M210, his2, cdc23-1E2*	This study
Bsp 129(1)	*h^{90}, leu1-32, ura4D18, his2, cdc23-M30*	This study
SPJ 541	*h^{90}, leu1-32, ura4D18, his2, ade6-M216*	This study
Bsp260	*h^{90}, leu1-32/leu1, ura4D18, ade6-M216, swi1-1, cdc23-M36*	This study
SPJ554	*h^{90}, leu1-32, ura4D18, ade6-M210, swi3-1*	This study
Bsp265	*h^{90}, leu1-32, ura4D18, ade6-M216, swi3-157, cdc23-M36*	This study
Bsp271	*h^{90}, ura4D18, ade6-M216, swi3-1, cdc23-M36*	This study
Bsp244	*h^{90}, leu1-32, ura4 D18, ade6-M210, his2, swi3-157*	This study
Bsp244	*h^{90}, ura4D18, ade6-M210, swi3-1*	This study
Bsp325	*h^{90}, ura4D18, ade6-M210, his2, swi3-146, cdc23-M36*	This study
Bsp146	*h^{90}, ura4D18, ade6-M210, swi3-146*	This study
Bsp381	*h^{90}, leu1-32, ade6-M216, ura4D18, his2 , cdc19-P1*	This study
Bsp382	*h^{90}, leu1-32 , ade6-M216, ura4D18, his2, mis5-268*	This study
Bsp383	*h^{90}, leu1-32 , ade6-M216, ura4D18, his2, nda4-108*	This study

Table S2. List of plasmids used in the study

#	Name of plasmid	Description	Source	Host
1	pART1			
2	pART1 *pol*α	*pol*α gene cloned in the vector pART1		
3	*nmt pol*α	*pol*α gene cloned in the vector pREP3		
4	*D-N pol*α	*pol*α^{D984N} gene cloned in the vector pREP3	T. Wang	
5	swi7-1/ *pol*α in BS	C-terminal Pol$\alpha^{swi7\text{-}1}$ cloned in XbaI – BamHI sites of BS	This study	DH5α
6	swi7-1/ *pol*α in pMALp2	C-terminal Pol$\alpha^{swi7\text{-}1}$ cloned in XbaI – PstI sites of pMALp2	This study	Codon-plus
7	Swi7-1 in pMALp2	C-terminal Pol$\alpha^{swi7\text{-}1}$ cloned in XbaI – PstI sites of pMALp2	This study	GroEL
8	GST-Cdc23(1)	Cdc23 cloned in EcoRI- BamHI sites of pGEX2T transformed in to DH5α	This study	DH5α
9	GST-Cdc23M36(1)	Cdc23-M36 cloned in EcoRI- BamHI sites of pGEX2T in DH5α	This study	DH5α
10	GST-Cdc23	Cdc23 cloned in EcoRI- BamHI sites of pGEX2T in BL21(DE3) Codon$^+$ cells	This study	Codon-plus
11	GST-Cdc23	Cdc23 cloned in EcoRI- BamHI sites of pGEX2T in BL21(DE3) GroEL cells	This study	GroEL
12	GST-Cdc23M36	Cdc23-M36 cloned in EcoRI- BamHI sites of pGEX2T in BL21(DE3) Codon$^+$ cells	This study	Codon-plus
13	GST-Cdc23M36	Cdc23-M36 cloned in EcoRI- BamHI sites of pGEX2T in BL21(DE3) GroEL cells	This study	GroEL
14	MBP-Polα7-1	C-terminal *pol*$\alpha^{swi7\text{-}1}$ cloned in XbaI – PstI sites of pMALp2 in codon Plus	This study	Codon-plus
15	pMALp2	Transformed into codon Plus	This study	Codon-plus
16	pMALp2	Transformed into XL1 Blue	This study	XL1 Blue
17	pMALp2	Transformed into GroEL host	This study	Codon-plus
18	MBP-Polα	Transformed into XL1 Blue	This study	XL1 Blue
19	Cdc23HA	*cdc23*$^+$ gene cloned into SalI-BamHI sites of pREP41HAN	This study	
20	Cdc23M36HA	*cdc23-M36* gene cloned into SalI-BamHI sites of pREP41HAN	This study	
21	Cdc23M30HA	*cdc23-M30* gene cloned into SalI-BamHI sites of pREP41HAN	This study	
22	Cdc23-1E2HA	*cdc23-1E2* gene cloned into SalI-BamHI sites of pREP41HAN	This study	
23	Cdc23E586A	*cdc23E586A* gene cloned into SalI-BamHI sites of pREP41HAN	This study	
24	Cdc23D587A	*cdc23D587A* gene cloned into SalI-BamHI sites of pREP41HAN	This study	
25	Cdc23D588A	*cdc23D588A* gene cloned into SalI-BamHI sites of pREP41HAN	This study	

Table S3 List of oligos used in this study.

Name of primer	Sequence 5'---------------3'	Supplier
cdc23 Rev Eco	ATGCATGAATTCTCAGGGAACTATTTCTAAGTCA	Sigma
cdc23 For Bam	ATGCATGGATCCATGCATGATCCCTTCATTGCA	Sigma
cdc233' For Bam	GTACGGATGGTCAGGGAACTATTTCTAAGGCA	Sigma
cdc23F-Sal1	ATGCATGTCGACATGCATGATCCCTTCATTGCA	Sigma
cdc23Rev E586A BamH1	ATGCATGGATCCTCAGGGAACTATTTCTAAGGCATCCGC	Sigma
cdc23Rev E587A BamH1	GCATGGATCCTCAGGGAACTATTTCTAAGTCAGCCTC	Sigma
cdc23D588A	GCATGGATCCTCAGGGAACTATTTCTAAGTCAGCCTC	Sigma
polα 5' BspH1	ATGCTCATGAGAAAGAGAACAAACGC	Sigma
polα 3' BamH1	GCGGATCCTCACGATGAAAATATCAGTCC	Sigma
MT1	AGAAGAGAGAGTAGTTGAAG	Sigma
MP	ACGGTAGTCATCGGTCTTCC	Sigma
MM	TACGTTCAGTAGACGTAGTG	Sigma

Figure S1. Defining the role of putative LAGLIDADG homing endonuclease and the restriction endonuclease domains in switching. A**,** ClustalX alignment of fission yeast Polα with known LAGLIDADG motif containing proteins. The putative conserved LAGLI-DADG motif is boxed. S.ce HO, *S.cerevisiase HO* gene; S. ce VMA1, *S. cerevisiae VMA1* gene; M.tb RecA, *M. tuberculosis RecA* gene; D. mb.I- Dmol, *D. mobilis* Intein*I-D mol* gene;T. lt. Vent pol, *T. litoralis* Vent polymerase gene; S.ce.I- SCEII, *S. cerevisiae* Intein*I-SceII*gene; S.p. polα, *S. pombe pol*α gene. Boxed regions indicate the LAGLI –DADG motif (green) and the numbers indicate the position of amino acid residues. **B,** A schematic diagram depicting the single copy integration of the mutated *polα* gene into the homologius site in the chromosomal copy of the *swi7-1* gene. **C,** The iodine-staining colony phenotype indicates mutant's switching defect. The *swi7-1* mutant's switching defect is complemented by the DADG domain mutations. Both the wt gene and all three mutated copies, A1, A2 and A1 plus A2, were cloned into the vector pART1. The light-staining phenotype of the *swi7-1* mutant, reflecting inefficient switching rate, is complemented by both wt as episomal copy and wt and all three mutated forms of *pol*α upon integration into *swi7-1* mutant as single copy, as indicated by increased staining of the transformant colonies. **D,** The $DX_{(6–30)}$[D/E]XK motif in Polα region-V. The sequence of *pol*α gene shows relevant three Asp(D) residues of D(E/E)XK motif. **E,** Iodine-staining complementation assay. The D(D/E)XK motif mutants were also cloned into the vector pART1. The mutated vectors were cleaved at the *Bgl*II site and integrated into the endogenous *swi7-1* mutant gene by homologous recombination. The vector and wild-type *pol*α transformants served as controls.

Figure S2. Polα displays no site-specific endonuclease activity towards *mat1M* locus *in vitro.* **A,B,** Time course analysis of treatment of *mat1M* DNA cloned into Bluescript with purified MBP-Polα (left panel) or Pol (right panel). **A,** supercoiled *mat1M* plasmid DNA; **B,** linear *mat1M* plasmid. **C,** sequence of *mat1M* and *mat1P* in the vicinity of the imprinting site, indicated by inverted arrows. **D,** sequence of readout of open circular *mat1M* DNA obtained from **A** after treatment with MBP-Polα and MBP. Inverted arrows indicate the expected position of break in sequence in case of a site-specific nick at the imprint.

Figure S3. The primase subunit mutant *spp1-4* is defective in directionality of mating-type switching. **A,** A schematic showing the specificity of the oligo pairs MT1-MP and MT1-MM in generating PCR products specific for *mat1P* (upper panel, purple) or *mat1M* (mauve), respectively. **B,** Agarose gel

electrophoresis pattern of products of multiplex PCR for DNA from the indicated strains. Sizes of PCR prodcuts for Plus and Minus mating type are indicated in base pairs (bp).

Figure S4. *cdc23* mutants show reduced mating-type switching. Strains with indicated genotype were grown on sporulating plates at 25ºC and 30ºC for 4 days and visualized by light microscopy.

Figure S5. No effect of *cdc23* mutant genes on switching in wt strain. wt h^{90} strain transformed with the indicated vectors were grown on plate lacking leucine for 3-4 days at 30ºC and stained with iodine. Numbers indicate efficiency of switching in the transformants.

Figure S6. Cumulative effect of the *swi7* (**A**), *swi1* and *swi3* (**B**) mutations with the *cdc23-M36* mutation on mating-type switching. Iodine-staining phenotype of colonies grown on minimal media at 30°C is shown. Numbers represent the level of switching. Synthetic lethality of *cdc23M36* with *swi7-1* (**C**), *swi1* and *swi3* (**D**) mutations on rich media. The indicated single and double mutant strains were streaked on rich (YEA) plates and grown at 30ºC.

Figure S7. SpMcm10/Cdc23 interacts with Swi1 but not with Swi3 *in vivo*. Coimmunoprecipitation experiment showing that Cdc23 interacts with Swi1 (A) but not with Swi3 (**B**) *in vivo*. Strains without tag or the indicated tags for Cdc23 (HA) and Swi1 (CBP) and Swi3 (myc) were used for immunoprecipitation followed by immunoblotting with the indicated antibodies.

Figure S8. No deleterious effect of *cdc23M36* mutation on the level of DNA Polα. (**A**) Equal amounts of proteins prepared from wt and *cdc23-M36* mutant grown at 25ºC and 30ºC were subjected to SDS-PAGE and western blotting with polyclonal anti-Polα (1:1,000) and anti-α-tubulin (1:2,000) antibodies. (**B**) Quantitation of the data shown in (**A**) done in triplicate.

Figure S9. MCM helicase complex subunit mutants are not imprint-defective at a semi-permissive growth temperature. (**A**) A schematic diagram depicting the MCM helicase complex migrating along the replication fork, recruits Mcm10/Cdc23 which, in turn, recruits the Polα-primase complex. (**B**) The indicated strains in h^{90} background were grown on PMA^{+} plates at 30°C for single colonies. Colonies were stained with iodine and photographed. The colonies of *mcm2*, *mcm5* and *mcm6* mutants give slightly darker iodine staining at 25ºC as they have high level of azygotic asci resulting from formation of diploids, possibly due to replication defect.

Figure S1

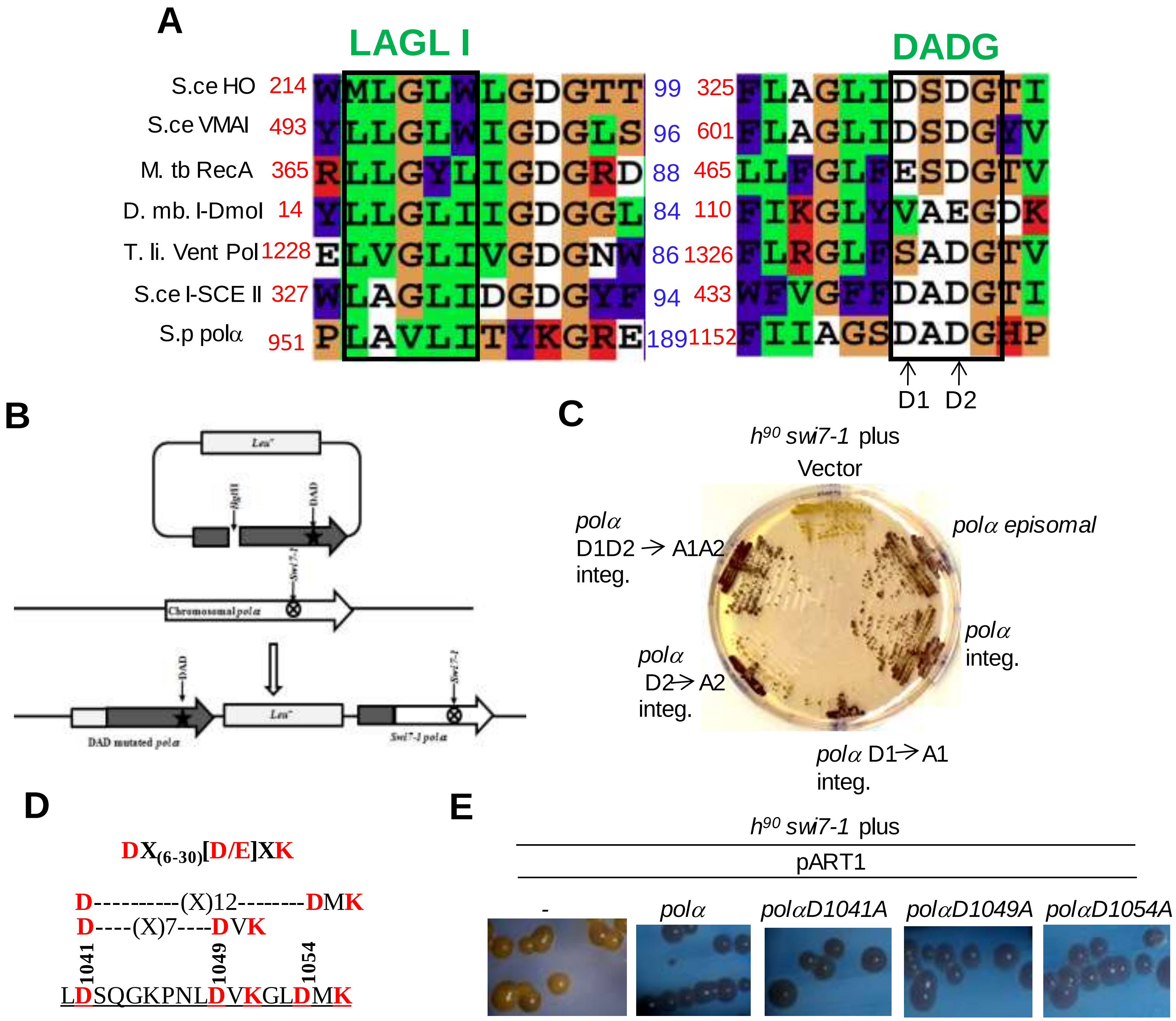
A
LAGL I
DADG
S.ce HO 214 WMLGLWLGDGTT 99 325 FLAGLIDSDGTI
S.ce VMAI 493 YLLGLWIGDGLS 96 601 FLAGLIDSDGYV
M. tb RecA 365 RLLGYLIGDGRD 88 465 LLFGLFESDGTV
D. mb. I-DmoI 14 YLLGLIIGDGGL 84 110 FIKGLYVAEGDK
T. li. Vent Pol 1228 ELVGLIVGDGNW 86 1326 FLRGLFSADGTV
S.ce I-SCE II 327 WLAGLIDGDGYF 94 433 WFVGFFDADGTI
S.p polα 951 PLAVLITYKGRE 189 1152 FIIAGSDADGHP
D1 D2
B
Leu+
BglII
DAD
Swi7-1
Chromosomal polα
DAD mutated polα
Swi7-1 polα
C
h90 swi7-1 plus
Vector
polα episomal
polα D1D2 → A1A2 integ.
polα integ.
polα D2 → A2 integ.
polα D1 → A1 integ.
D
DX(6-30)[D/E]XK
D----------(X)12---------DMK
D----(X)7----DVK
1041
1049
1054
LDSQGKPNLDVKGLDMK
E
h90 swi7-1 plus
pART1
-
polα
polαD1041A
polαD1049A
polαD1054A

Figure S2

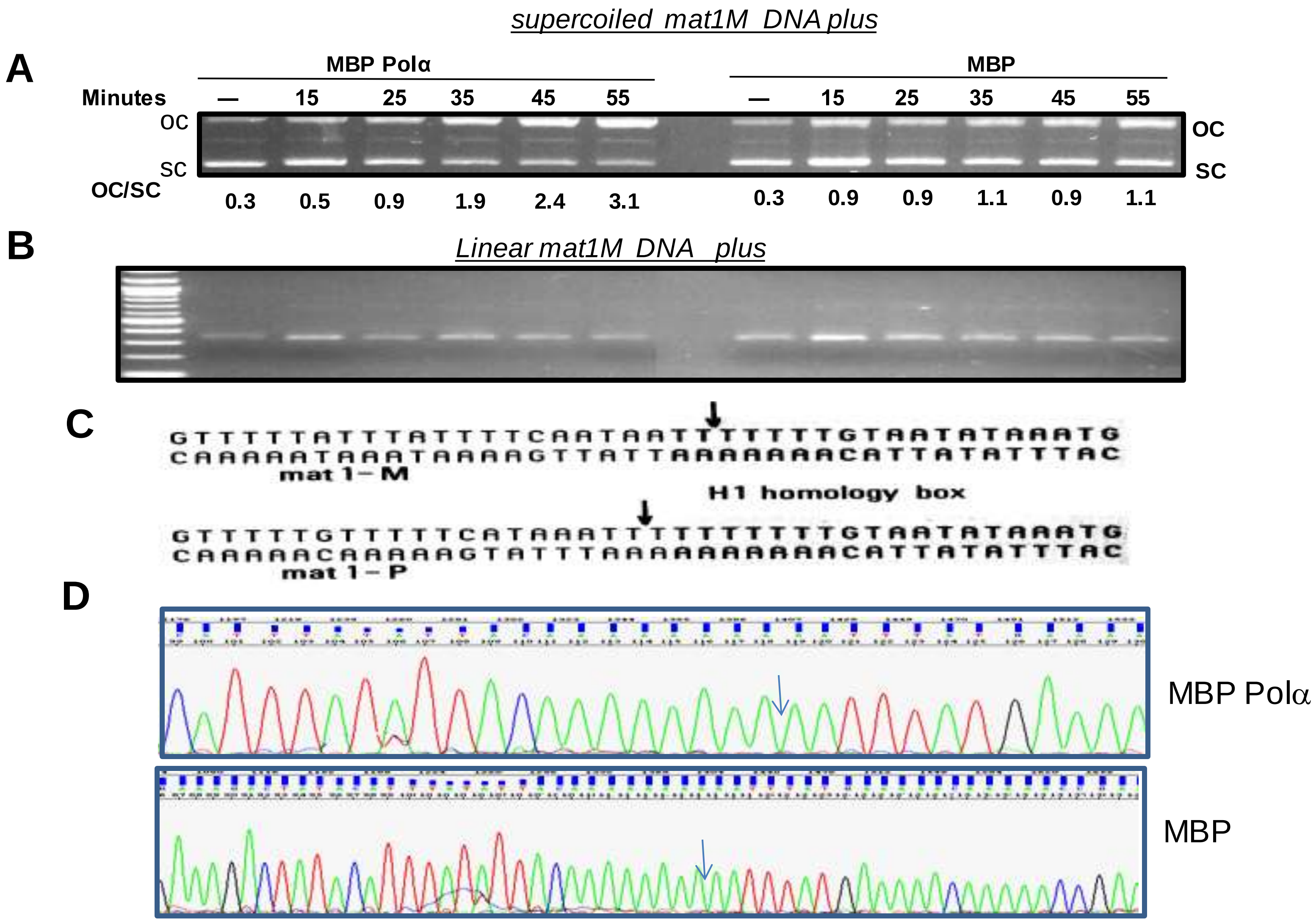
supercoiled mat1M DNA plus
A
MBP Polα
MBP
Minutes
—
15
25
35
45
55
OC
SC
OC/SC
0.3
0.5
0.9
1.9
2.4
3.1
0.3
0.9
0.9
1.1
0.9
1.1
B
Linear mat1M DNA plus
C
GTTTTTATTTATTTTTCAATAATTTTTTTTGTAATATAAATG
CAAAAATAAATAAAAGTTATTAAAAAAAACATTATATTTAC
mat1-M
H1 homology box
GTTTTTGTTTTTCATAAATTTTTTTTTTTGTAATATAAATG
CAAAAACAAAAAGTATTTAAAAAAAAAAACATTATATTTAC
mat1-P
D
MBP Polα
MBP

Figure S3

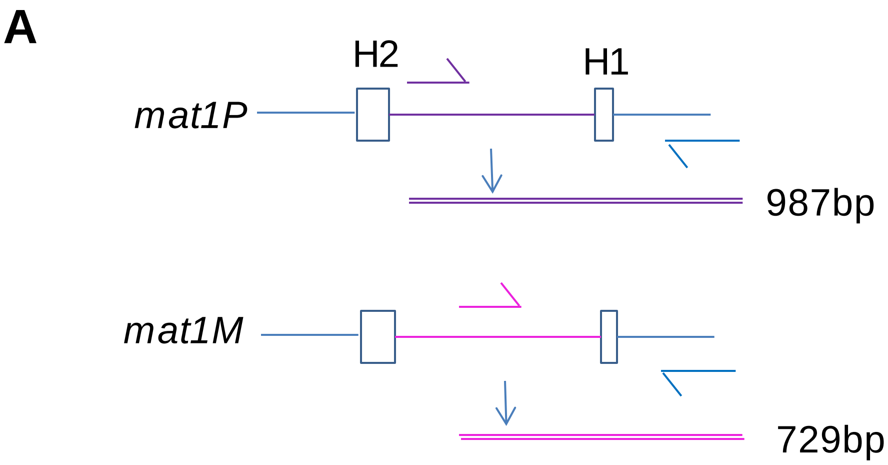
A
H2
H1
mat1P
987bp
mat1M
729bp

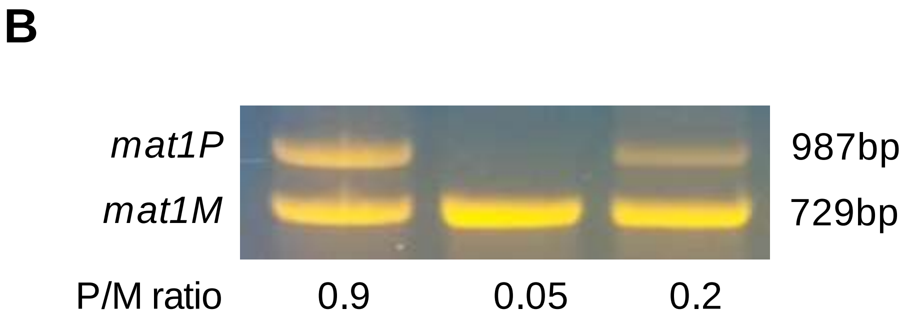
B
mat1P
mat1M
987bp
729bp
P/M ratio
0.9
0.05
0.2

Figure S4

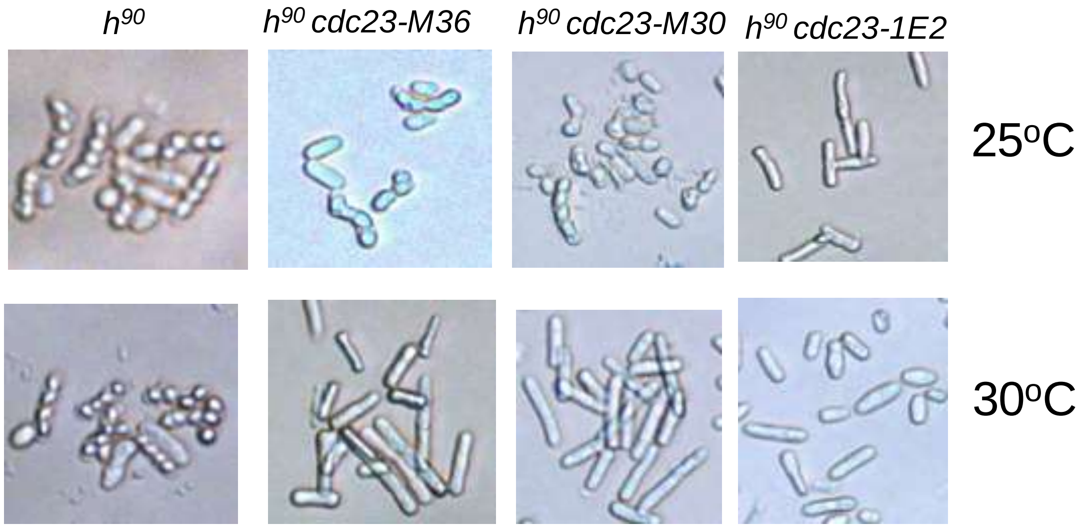

<u>*h^{90} cdc23-M36 plus*</u>

Vector: 86.5% *cdc23-D588A: 81.5%*

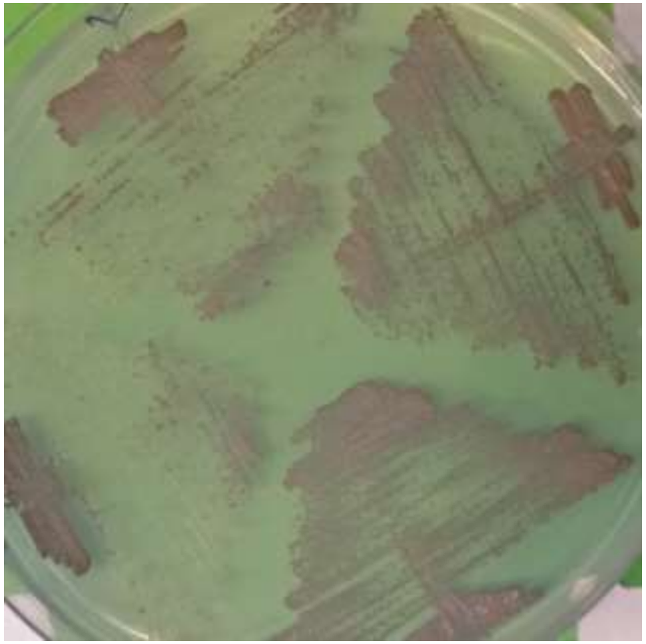

Cdc23: 84.9% *cdc23-M36: 89.6%*

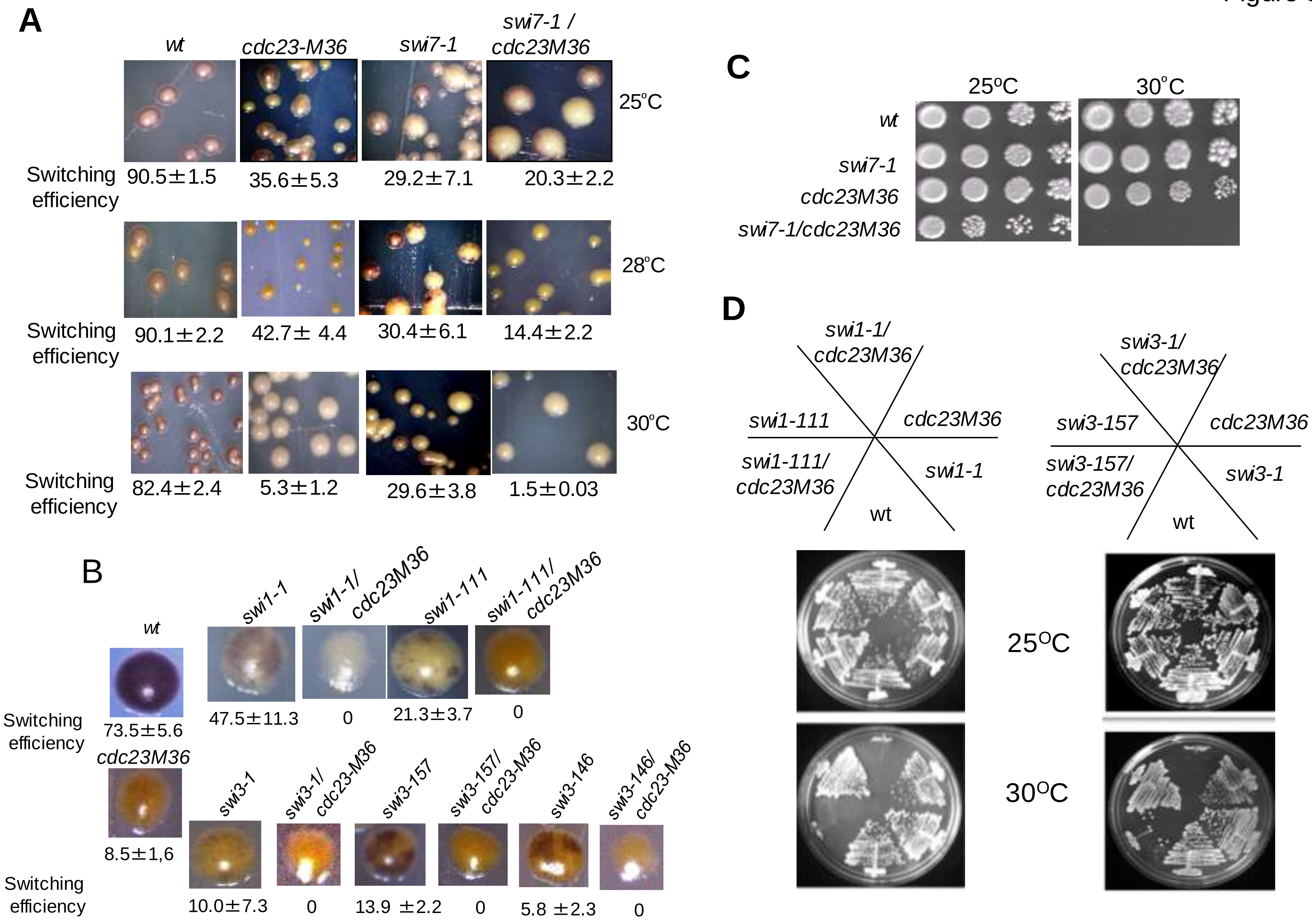
A
wt
cdc23-M36
swi7-1
swi7-1 / cdc23M36
25°C
Switching efficiency
90.5±1.5
35.6±5.3
29.2±7.1
20.3±2.2
28°C
Switching efficiency
90.1±2.2
42.7± 4.4
30.4±6.1
14.4±2.2
30°C
Switching efficiency
82.4±2.4
5.3±1.2
29.6±3.8
1.5±0.03
B
wt
swi1-1
swi1-1/ cdc23M36
swi1-111
swi1-111/ cdc23M36
Switching efficiency
73.5±5.6
47.5±11.3
0
21.3±3.7
0
cdc23M36
8.5±1,6
swi3-1
swi3-1/ cdc23-M36
swi3-157
swi3-157/ cdc23-M36
swi3-146
swi3-146/ cdc23-M36
Switching efficiency
10.0±7.3
0
13.9 ±2.2
0
5.8 ±2.3
0
C
25°C
30°C
wt
swi7-1
cdc23M36
swi7-1/cdc23M36
D
swi1-1/ cdc23M36
swi1-111
cdc23M36
swi1-111/ cdc23M36
swi1-1
wt
swi3-1/ cdc23M36
swi3-157
cdc23M36
swi3-157/ cdc23M36
swi3-1
wt
25°C
30°C

Figure S7

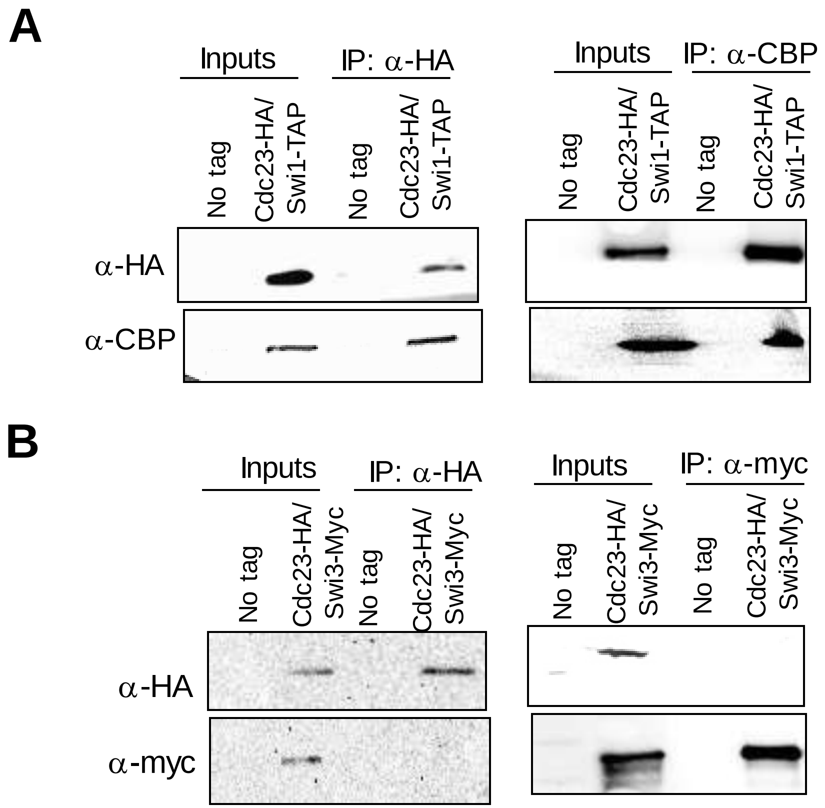
A
Inputs
IP: α-HA
No tag
Cdc23-HA/ Swi1-TAP
No tag
Cdc23-HA/ Swi1-TAP
α-HA
α-CBP
Inputs
IP: α-CBP
No tag
Cdc23-HA/ Swi1-TAP
No tag
Cdc23-HA/ Swi1-TAP
B
Inputs
IP: α-HA
No tag
Cdc23-HA/ Swi3-Myc
No tag
Cdc23-HA/ Swi3-Myc
α-HA
α-myc
Inputs
IP: α-myc
No tag
Cdc23-HA/ Swi3-Myc
No tag
Cdc23-HA/ Swi3-Myc

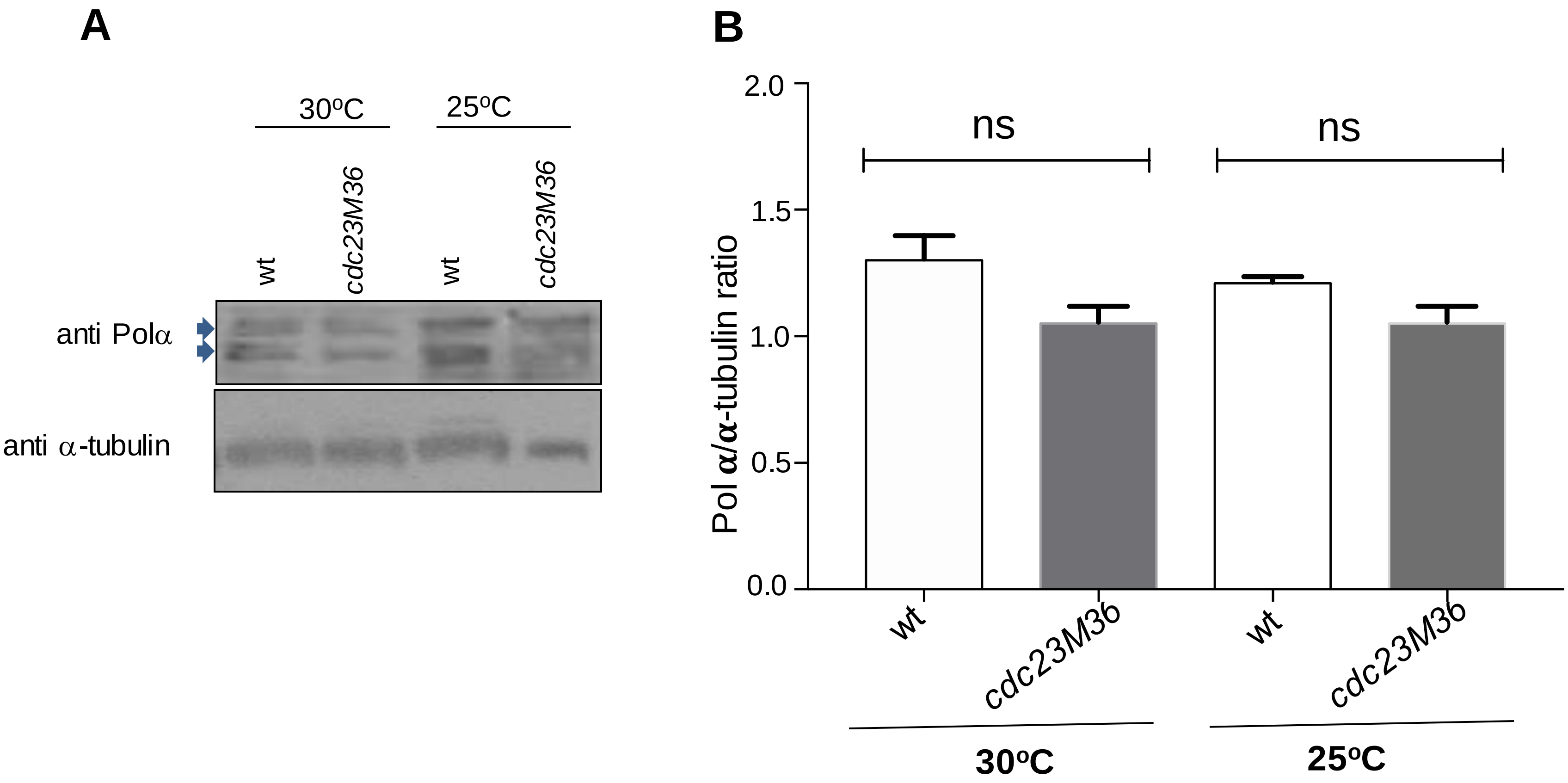
A
30ºC
25ºC
wt
cdc23M36
wt
cdc23M36
anti Polα
anti α-tubulin
B
Pol α/α-tubulin ratio
2.0
1.5
1.0
0.5
0.0
ns
ns
wt
cdc23M36
wt
cdc23M36
30ºC
25ºC

Figure S9

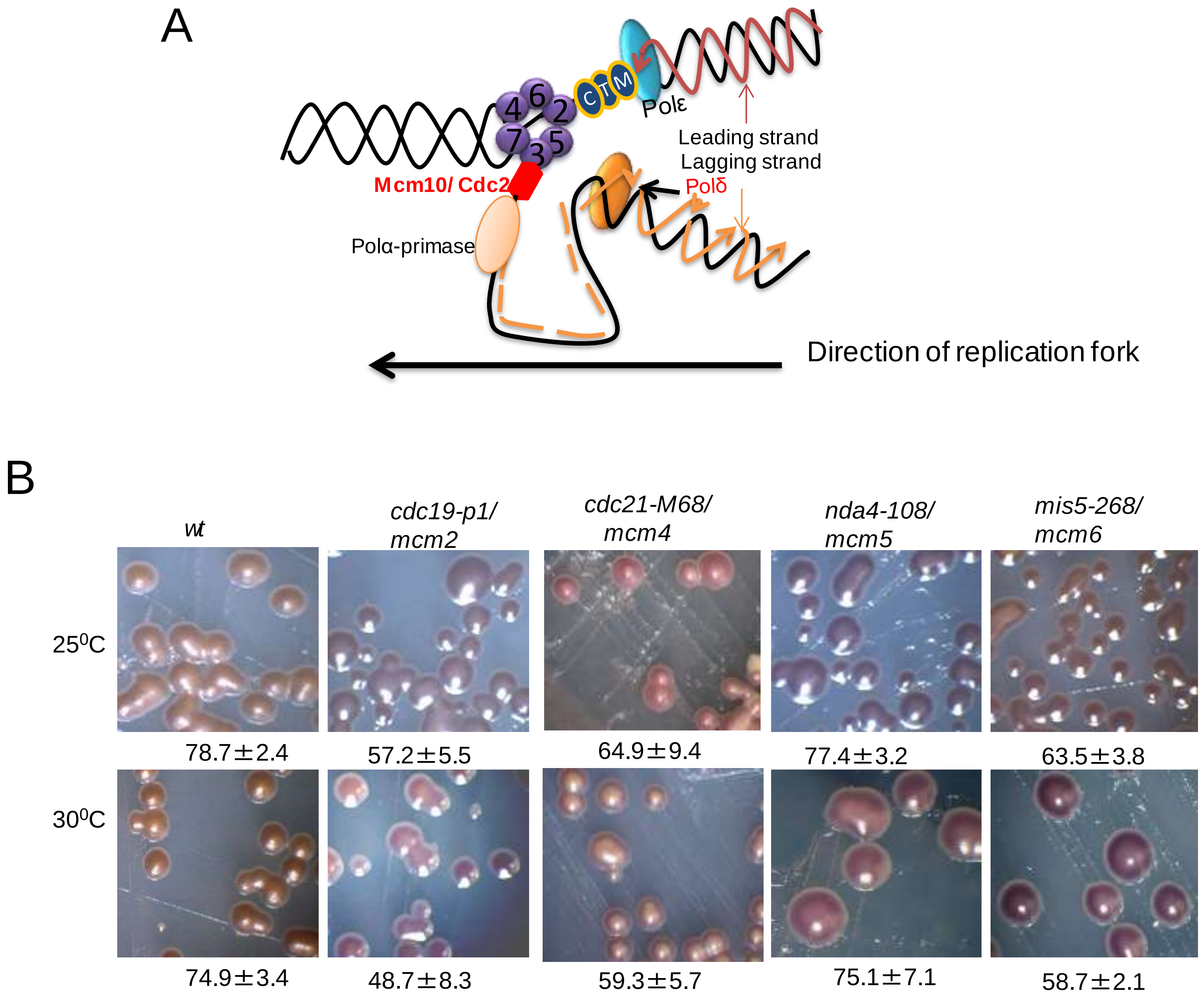
A
C
T
M
Polε
Leading strand
Lagging strand
Polδ
Mcm10/ Cdc23
Polα-primase
Direction of replication fork
B
wt
cdc19-p1/ mcm2
cdc21-M68/ mcm4
nda4-108/ mcm5
mis5-268/ mcm6
25⁰C
30⁰C
78.7±2.4
57.2±5.5
64.9±9.4
77.4±3.2
63.5±3.8
74.9±3.4
48.7±8.3
59.3±5.7
75.1±7.1
58.7±2.1
